# Supplementary figures and images for: Resilience of Canola to Plasmodiophora brassicae (Clubroot) Pathotype 3H under Different Resistance Genes and Initial Inoculum Levels
Source: Plants (Basel). 2024 Jun 2;13(11):1540. doi: 10.3390/plants13111540 (PMC11174560; doi:10.3390/plants13111540)

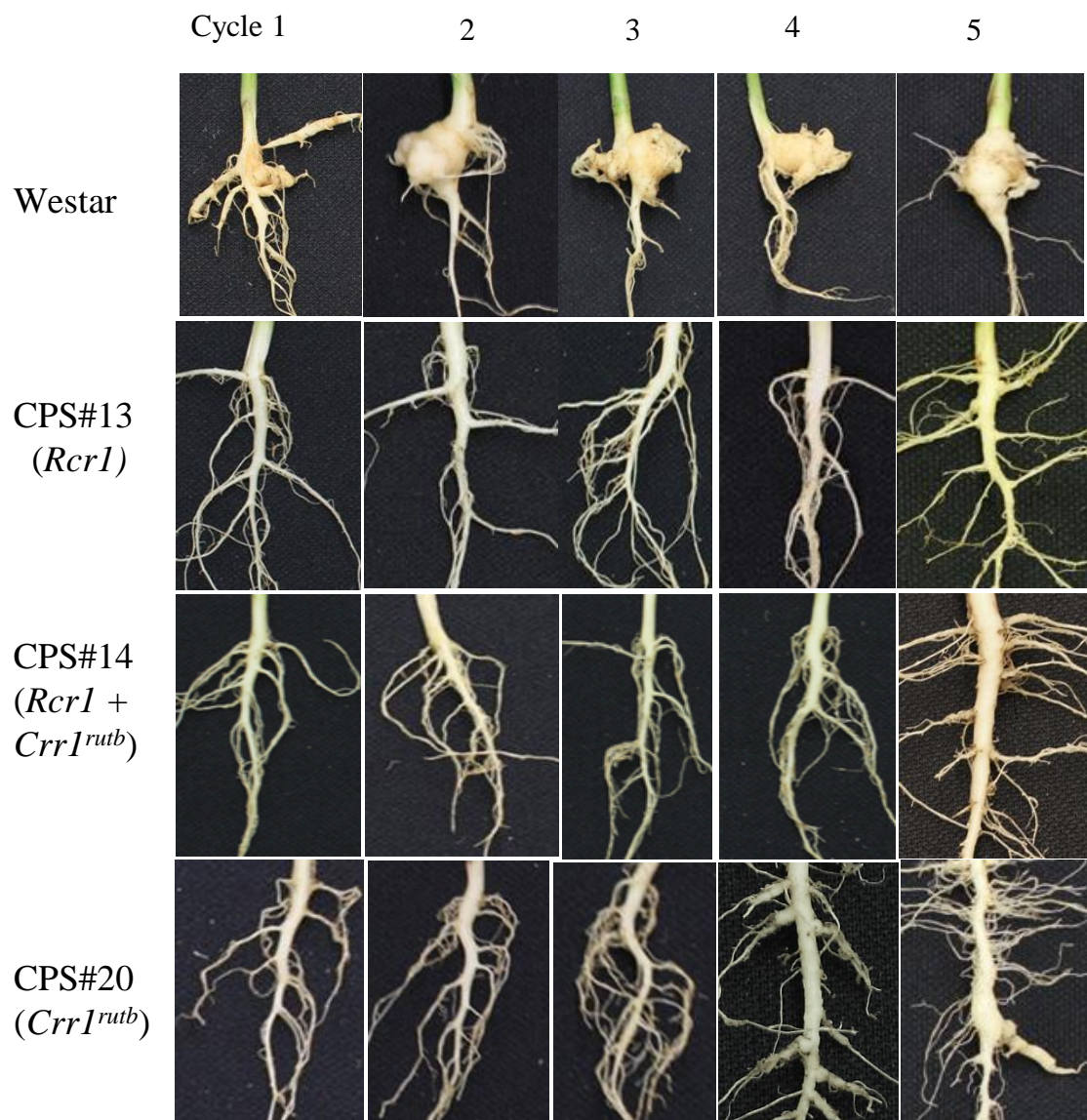

Supplementary Figure S1

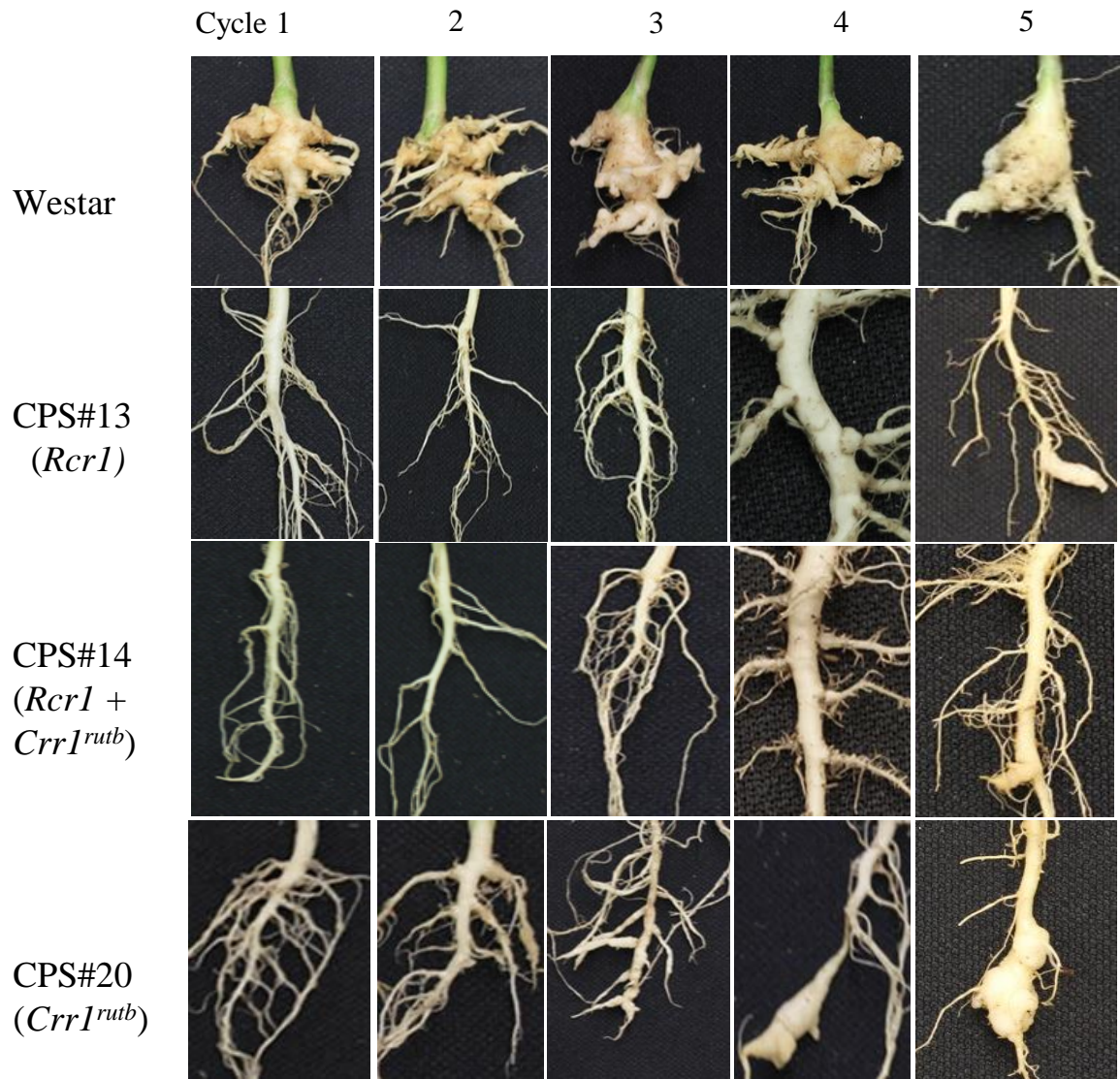

Supplementary Figure S2

Supplement: Supplementary file 1 [file plants-13-01540-s001.zip › plants-3001601-supplementary.pdf]
